# Clonal Propagation of Walnuts (*Juglans* spp.): A Review on Evolution from Traditional Techniques to Application of Biotechnology

**DOI:** 10.3390/plants11223040

**Published:** 2022-11-10

**Authors:** Kourosh Vahdati, Rasoul Sadeghi-Majd, Adriana F. Sestras, Ricardo Julián Licea-Moreno, Augusto Peixe, Radu E. Sestras

**Affiliations:** 1 Department of Horticulture, College of Aburaihan, University of Tehran, Tehran 3391653755, Iran; 2 Department of Forestry, University of Agricultural Sciences and Veterinary Medicine, 400372 Cluj-Napoca, Romania; 3 Department of Biotechnology, Micropropagation Unit, Bosques Naturales S.A., 28108 Madrid, Spain; 4 MED–Mediterranean Institute for Agriculture, Environment and Development, Department of Plant Science, School of Science and Technology, University of Évora, Pólo da Mitra, Ap. 94, 7006-554 Évora, Portugal; 5 Department of Horticulture and Landscape, University of Agricultural Sciences and Veterinary Medicine, 400372 Cluj-Napoca, Romania

**Keywords:** acclimatization, adventitious root formation, grafting, in vitro culture, layering, vegetative propagation, walnut

## Abstract

Walnuts (*Juglans* sp.) are allogamous species. Seed-derived plants are not always superior to the selected parent. Clonal propagation of selected stock plants is an essential requirement for the clonal fidelity of the descendants and to maintain their genetic structure. Selection of the desired plant is realized only after reaching maturity, and characterizing and evaluating the performance of adult trees require a long time. Clonal propagation methods ensure proper transmission of characters to descendants and can be used effectively in breeding programs. The commercialization of a cultivar or rootstock depends on the success of vegetative propagation. Walnuts, like other tree species, are recalcitrant to conventional vegetative propagation methods and even non-conventional in vitro culture (micropropagation). Elucidation of factors determining the success of cloning of desired plants would contribute to understanding current limitations for most genotypes of *Juglans*. We outline the role of grafting and cuttings and stool layering, as well as in vitro culture on walnut multiplication. These techniques are, in practice, entirely different; nevertheless, they are affected by common factors. The incompatibility of stock-scion and the reduced ability of stem cuttings to root are the main bottlenecks for grafting and cutting, respectively. Genotype, age, and physiological status, reinvigoration or rejuvenation-treatment of donor plant, period of harvesting and processing of explants critically affect the results of methods followed. The in vitro culture technology is the most suitable for walnut cloning. This also has constraints that affect commercial propagation of most desired genotypes. We describe comprehensive results and synthesis in this review on the asexual reproduction of walnuts, providing a better comprehension of the limiting factors and the ways to overcome them, with direct implications on commercial propagation and the releasing of outstanding genotypes.

## 1. Introduction

Walnuts are mostly propagated by seeds. The seed-derived offspring of walnuts is highly heterozygous. It usually leads to the obtaining of a low quantity of propagules and reduced quality of production, as well as long juvenility periods, thus delaying the return on investments [1]. Nowadays, plants from seeds are only used for rootstock production [2]; however, the high variability of these materials reduces their quality. Although the easiest traditional method for clonal propagation of walnuts is grafting, it is a labour-intensive and time-consuming method driven towards produce high-cost plants [3]. The complexity of this method causes high percentages of failure, contributing to higher unit costs. The success of walnut grafting is highly dependent of several factors, from internal and environmental ones to those derived from human expertise [4].

Cuttings and stool layering are also used for walnut propagation. Juvenility and rejuvenation of stock plants, propagule vigour, time of harvest, stock plant manipulation and rooting substrate affect the success of cutting and stool layering methods [5,6,7,8]. Cuttings and stool layering are an alternative to grafting; however, they are less widely used methods due to their low success rate.

Micropropagation is a suitable method for propagation on a large scale [9,10,11,12,13,14]. Although its success depends on numerous intrinsic and extrinsic factors, the formation of adventitious roots (ARs) is the most critical. Adventitious root formation (ARF) in walnuts is a complex process that usually includes three phases [15,16,17]: (1) induction of the presence of hormones, producing the biochemical and physiological transformations needed to reach rooting capacity; (2) initiation, characterized by cell division and establishment of root primordia, and (3) root expression, during which root meristems develop and connect to the vascular bundles. Thus, the obtaining of adventitious roots can be performed following one of two pathways: (1) direct organogenesis in which established cell types such as cambium, cortex, pericycle or vascular bundles re-differentiate, or (2) indirect formation from callus tissue [18] there are also other relevant factors, such as hormonal imbalance, mature tissues unable to respond to auxin, etc. These processes drive the formation of root primordium, which is regulated by different internal and external factors. Besides the environmental conditions (temperature, humidity or light) and treatments applied to the mother plants, several physical and chemical factors (oxygen, pH, enzymes, phytohormones, polyamines, carbohydrates or phenolic compounds) can affect AR initiation in walnut [19].

Along with ARF, the success of commercial micropropagation programs is dependent on the recalcitrance of most of the genotypes to be in vitro cultured. Although multiplication rates (MR) of the established clones do not represent a great limitation for commercial micropropagation, the increasing of MRs undoubtedly contribute to the improvement of rooting and survival during acclimatization, which would reduce the production costs of in vitro plants.

We present an evaluation of reports and methods applied to clonal propagation of walnuts and the factors that affect the success/failure, from the most traditional propagation methods to the most recent and promising techniques, such as micropropagation. The comprehensive information presented here may help diverse professionals and experts in improving the commercial propagation of most genotypes of walnuts.

## 2. Walnut Grafting

### 2.1. Traditional Walnut Grafting: An Overview

It is well documented that walnuts are more difficult to perform grafting/budding on than other fruit trees [1,20,21,22]. Temperature, relative humidity, physiological condition of the rootstock, scion type, water status of the scion and rootstock, time of grafting, grafting methods, xylem sap exudates, hormones and phenolic compounds can affect walnut grafting success [4,21,23,24,25,26].

#### Factors Affecting Walnut Grafting

- Environmental factors and determining the best grafting time

At different times of grafting, due to different temperatures and relative humidity, the success of grafting varies; thus, part of the research has been done in controlled and semi-controlled conditions [25,27]. Suitable temperature (26–27 °C) and relative humidity (80–95%) are very important to optimize grafting because these factors are essential for callus formation at the graft site. From late winter to early spring, there is good humidity and temperature for grafting but the walnut grafting success is limited due to root pressure. It has been shown that heavy irrigation or rainfall before and after grafting can increase root pressure and consequently reduce graft success [1,28]. Contradictory reports have examined the effect of irrigation on walnut grafting success. Whereas some reports showed the negative effect of irrigation or rainfall before and after grafting [1,28], some others have revealed a positive effect, through “reducing the temperature and increasing the relative humidity of the environment”, or no significant effect [24]. However, in conditions where the soil water potential is high and the transpiration rate is low, the root pressure is more likely to occur [1]. Data suggest that a flush of bleeding may start about 7 days after a temperature spike; a rapid increase in temperature, followed by a rapid drop within a few days [1,28]. However, there are several methods to control the root pressure and to increase the walnut grafting success, such as using head back rootstocks two to three inches above the grafting site one to two weeks earlier than the grafting time, making cuts in the rootstock through the bark and into the wood at several places below the graft site, pruning roots with a plow, transplanting plants into the second nursery, side grafting, covering the graft site with materials such as sawdust to absorb the sap, etc. [22,27,28,29,30]. In a report, Sharma and Joolka [31] compared patch budding in Solan (a city in the Indian state of Himachal Pradesh, India, with an altitude of 1600 m and where the temperatures hardly rise more than 35 °C) at different times (2, 10, 18 and 26 July and 3, 11 and 19 August) and found that the optimal time for budding was July 2 with 80% bud take. Of course, as is well documented, the proper time for grafting varies in different regions because plants are in different phenological stages.

Significant effects of grafting techniques on walnuts have been reported [32]. Different types of grafting, such as side stub, omega, whip and tongue, saddle or V grafting, and also patch, chip and shield budding and top working have been studied by various researchers [21,24,25,29,33,34,35,36]. Different grafting methods due to differences in the physical overlap between the cambial layers of the rootstock and the scion have different results; Sadeghi Majd et al. [21] reported that the V grafting method with the Carlo A. Manares Co. Machine (Italy) has more success. Studies show that a certain grafting method is not the best grafting method for all regions, and success is strongly influenced by the skill of workers. At present, patch budding is the most important method for walnut grafting. In a report where omega grafting was compared with side stub and whip grafting, the highest callus quality, graft take and grafting survival were obtained with the omega method, followed by side stub and whip grafting [29,33]. Rezaee et al. [30] showed that modified bark grafting performed in mid-April had the highest grafting success and survival percentage (100 and 96.3%, respectively) compared to cleft and whip tongue grafting. A high rate of patch budding success under controlled conditions was reported [25,27]. Ebrahimi et al. [27] obtained the highest graft success by patch budding with 91% compared to shield (31.1%) and chip (19.1%). Two bench grafting methods, including whip and tongue techniques, are very successful [29,37]. It is quite clear that high temperature during grafting has a detrimental effect on callus growth [22,25,27,30]. Insufficient callus formation at the graft site by scion and rootstock is a major cause of graft failure in walnuts, and it has been shown that highly turgid cells are more likely to proliferate calluses than those in wilting conditions [33,38] therefore, in some studies, the hot callus method (local heating of the graft union with a hot callusing pipe or electric cable, etc., to achieve a temperature of 26–27 °C) has been used to provide the appropriate temperature at the graft site and also the use of moist covering at the graft site to increase the moisture required for callus formation [37,38,39]. Ghamari Hesabi et al. [40] stated that for budding, the rootstock must be strong and well-watered until the bark is easily separated from the wood. Therefore, by controlling the environmental conditions and performing the grafting/budding at a suitable time [41], the success of the grafting/budding is guaranteed.

- Physiological and genotype-dependent factors

Vigorous rootstocks (two-year-old) cause higher graft success when compared to 1-year-old seedlings (i.e., less vigorous); this is due to the faster healing in the graft site at stronger rootstocks, as they have a higher quality of callus produced at the graft site [42,43,44,45]. But it is reported that low vigor rootstocks have lower root pressure compared to semi- and high-vigor seedlings and therefore have more successful grafting.

Callus formation and vascular differentiation at the grafting site are important results of hormones such as the auxin and cytokinins [46,47,48,49]. Auxin indole-3-acetic acid (IAA) released from the rootstock and scion induces callus formation, cambium development and differentiation of vascular tissue and vascular bridge between rootstock and scion [50,51,52,53,54,55,56,57]. Despite the phenolic compound that may accumulate in small amounts at the grafting site and have no significant effect on the success, these concentrations could be expected to increase to lethal levels after evaporation and have a bad effect in creation of the callus bridge. The most important phenolic material in walnut is juglone (5-hydroxyl-1, 4-naphthoquinone); due to its chemical reactivity in the formation of free radicals and damage, it has a destructive effect on tissues through necrosis, apoptosis and cell death in grafting sites [58,59,60]. Due to the low level of phenolic compound in early spring and late-summer, grafting success is high when compared with early summer [61]. In addition, a high level of phenolic compounds above the graft site prevents callus formation and tissue browning after a cut and has a detrimental effect on the differentiation of vascular tissues by imposing limitations on auxin transfer [52,62,63]. 

Rootstock and scion genetics have been reported to be effective in walnut grafting success, although the strength of this effect ranges from very minor [21,29,64] to major [65,66]. The different values attributed to grafting success could be explained by the physiological status of the mother stock (scion), rather than its genetic structure [21]. The better ripeness and quality of the scions, which contain higher soluble sugars, starch and C/N ratio led to the higher grafting survival percentage [37,38,39]. The moisture content of the scion should not be below its critical level (38.48%) because this has a destructive effect on callus formation [49]; so, with proper irrigation and prevention of water stress on mother trees and the covering of the graft site with moist materials, graft success can increase.

### 2.2. Hypocotyl, Epicotyl and Mini-Grafting of Walnut

The grafting of young walnut seedlings has been introduced as a simple and fast procedure for Persian walnut propagation. These methods are known as hypocotyl grafting, epicotyl grafting and mini-grafting [26,34,67,68,69,70,71]. In epicotyl grafting, walnut seeds are planted in containers or pots filled with a lightweight substrate (for example, coco-peat and perlite at 1:1 *v*/*v* ratio). Four weeks after germination, 1–2 cm above the epicotyl, the rootstocks are cut and grafted using the v method, with the dormant scions used containing two buds 5–8 cm long, and then the grafting point is covered with moisture-absorbing cover material and tied with parafilm tape. Gandev and Arnaudov [68] obtained a 73% grafting success rate with this method. Ráufi et al. [34] obtained a 61% grafting success rate, and Suk-In et al. [72] showed 78.9% survival rate, on average. The important factor is to keep the relative humidity around 80%. Another method of early grafting is the hypocotyl method, which is performed on seedlings less than 5 months old. In the experiment conducted by Soleimani et al. [67], three months after seed germination, the seedlings were grafted using the hypocotyl method and kept in a greenhouse at a temperature of 26 °C and a relative humidity of 90–95% for one month.

Another method, called mini-grafting, is performed under controlled conditions, and it is possible to graft two- to three-month-old to one- to two-year-old seedlings. In this method, which is often done in a V–shape after the grafting, the grafted plants are covered by clear plastic cups to avoid losing moisture. In an experiment, the highest percentage of graft-take was obtained by using 50 mg/L IBA + 80 mg/L BA [69]. Balanian [70] compared the success of minigrafting on two types of green and softwood rootstocks and reported that graft site repair was faster on green rootstocks but the graft takes on softwood rootstocks was higher than green rootstocks (73% vs. 40%, respectively). However, the use of compounds such as polyvinyl pyrrolidone (PVP), ascorbic acid and citric acid as antioxidants reduces the oxidation of phenolic compounds and tissue necrosis at the grafting point [70,73]. Despite the practicality of these methods, traditional methods of grafting are still preferred for various reasons, including survival, and these methods still need optimization.

### 2.3. Walnut Micro-Grafting

Despite the availability of clonal rootstocks, there are problems in the production of grafted walnut plants by traditional grafting methods due to various reasons, including long, seasonal, and time-consuming process [74]. Micro-grafting, which is mostly performed under aseptic conditions, includes the placement of a maintained scion onto an in/ex vitro-grown rootstock and can minimize these problems [47]. In this method, scions and rootstocks are produced in two in/ex vitro conditions. In one of these methods, scion and rootstock are taken from plant material that grow under aseptic conditions and grafted under these conditions, and in another method, grafting may be performed during the transition from in vitro to ex vitro, either during or after the acclimatization phase of the rootstock. This technique has important potential effect, including the production of virus-free plants, the study of scion–rootstock interactions and long-distance signaling, and virus indexing [75]. In an experiment, seedling rootstocks were grafted in ex vitro conditions by using scions from three Persian walnut (*J. regia*) cultivars produced in vitro, and the result was reported to be a 90% rate of graft survival [76], The scion genotype, the seedling age, the use of plant growth regulators and speed implementation of the technique to avoid dehydration of the in vitro-produced explants [74] were considered as the important factors in the grafting success.

In an experiment, cuttings of a cultivar (‘Chandler’) and a rootstock (‘Vlach’) were produced and multiplied under in vitro conditions for at least 10 subcultures. The rootstocks were prepared from the bottom part of shoots; but scions, for cleft grafting, were prepared from the upper part of shoots with 30 to 40 and 20 to 50 mm height, respectively, and rootstocks were selected from explants from the in vitro root expression medium vs. scions that were prepared from explants grown in in vitro multiplication medium [74]. They reported that simultaneous rooting and micrografting steps are possible in walnut explants obtained from in vitro cultured adult explants, and it was also reported that the presence of leaves on the rootstocks and scion improves the rootstock rooting and grafting success rate (70% in explants with the presence of leaves on scions and rootstocks). Auxins are known to be key regulators of cell proliferation and differentiation, activation of cambium proliferation and regulation of vascular differentiation, and young leaves and apical meristems are the primary and key sites of auxin biosynthesis. The use of seedling rootstocks is recommended in producing virus-free plants or testing long-distance signaling, but when the goal is vegetative propagation, the use of clonal scions and rootstocks is mandatory. However, compared to using traditional rooting and grafting procedures that take two to three years, using this method can reduce the time needed for plants ready for orchard planting to less than one year.

## 3. Propagation by Cuttings and Stool Layering

### 3.1. An Overview

Continuity of a sclerenchymatous layer surrounding the phloem and oxidation of phenolic compounds at the wounding site are the main factors in reducing rooting and the emergence of roots in walnut [77]. Published attempts at rooting cuttings and layering, using a variety of treatments, show that genotype is one of the crucial factors influencing outcome [5,77,78,79].

### 3.2. Factors Affecting Rooting of Cuttings and Layers

#### 3.2.1. Juvenility and Rejuvenation of Stock Plants

The age of the donor plant, which negatively affects rooting capacity, was reported by Aumond et al. [80]. Rejuvenation of mature trees, the inducement of plants to regress from maturity to the juvenile state, increases adventitious root (AR) formation [6]. Mature and juvenile trees have different phenotypes, organizational structure and physiology, and rejuvenation can increase the activity of esterases and peroxidases [6]. As a tree ages, the bottom and older parts remain juvenile, while the more recently grown branches are mature. Therefore, in some reports, seedlings or trees are severely pruned (coppiced) or cut down to produce immature shoots. Also, Guan et al. [81] reported easier rooting of juvenile cuttings versus mature and woody tissues due to differential auxin biosynthesis and perception. However, the molecular and biochemical mechanisms are not yet completely clear.

#### 3.2.2. Propagule Vigor

Comparison of easy-to-root (*Eucalyptus grandis* W.Hill ex Maiden) and hard-to-root (*E. globulus* Labill.) eucalyptus showed that delay in xylem development, with less lignification in *E. globulus* compared with *E. grandis*, indicates less activity of the cambium layer as an important site for AR development [82]. Results showed that *E. grandis* (easy-rooting) microcuttings have larger xylem area than *E. globulus* (hard-rooting) and also Superoxide dismutase (SOD) and that both cell-wall bound and soluble guaiacol peroxidase (GPRX) activities were indicative of rooting capacity [82].

In some herbaceous plants, reduction of shoot growth by application of growth inhibitors such as triazole also increased rooting [7]. Vigorous layered shoots produced more callus, and the reduction in rooting rate in the more vigorous trees may be due to greater gibberellin content, greater lignification, higher wood density or rigidity of the sclerenchymal ring [5].

Vigor can also affect the origin of adventitious roots (Ars) in walnut. In low-vigor rootstocks, most ARs are produced internally from vascular cambium, while in other cases, ARs originate directly from callus [20].

#### 3.2.3. Time of Collecting Propagule

Cutting time and wood type (hard-wood, semi-hard-wood and soft-wood cutting) of cuttings have a great impact on the success of rooting walnuts and other species. Collection dates of cutting shoots (one- or two-year-old shoot cuttings of walnut) in November, December, January and February were correlated with length of time required for callus formation following IBA application. Older shoots exhibited delayed callus formation, and January cuttings had the best callus morphology, but the final callus rate was not significantly affected by the age or IBA treatment [77]. Tajbakhsh et al. [8] found 81% of walnut hardwood cuttings taken in December callused, vs. 67% of cuttings taken in March.

When semi-hardwood and hardwood cuttings were used to root ‘Paradox’ clone ‘MX200’, rooting percentages were 43% and 14%, respectively. In contrast, hardwood cuttings of Paradox clone ‘AZ025’ produced more roots (13%) than semi-hardwood cutting (0%) [83]. Effect of collection time (spring vs. summer) and hormonal treatment (0, 29 or 62 mM K–IBA and 0, 34 or 74 mM IBA) on rooting of softwood (collected in June and July) and hardwood (collected in March, April and May) cuttings of *J. cinerea* were investigated [5]. The greatest rooting percentages were obtained with 74 mM IBA and 62 mM K–IBA, regardless of the collection time. Softwood cuttings rooted best when taken in June (current season’s first flush of new growth or softwood growth 40 cm or greater) and treated with 62 mM K–IBA (77%) or 74 mM IBA (88%). However, for hardwood cuttings, rooting was greatest for those taken in mid-May (branches flushed out), 22% with 62 mM K–IBA and 28% with 74 mM IBA. For *J. regia*, more herbaceous cuttings obtained from mother trees by severe pruning for several years can facilitate rooting due to the non-lignification of strong tissues, such as sclerenchyma, and the production of phenolic compounds [22]. Accordingly, we suggest that due to the diversity in the phenology of walnut cultivars (different phenology of genotype and cultivars at the same time), the rooting percentage is different among cultivars. In any case, the definitive answer regarding differences in ARF of cultivars are not clear; further, due to the lower sclerenchyma tissue layer in younger cuttings, along with the low number of phenolic compounds in early spring (when softwood cuttings are taken), the rooting of these cuttings has been reported more than others.

#### 3.2.4. Stock Plant Manipulation

Characteristics related with donor plant, which include plant health, vigor, nutritional status, growth stage, organ and tissue selected as initial explant influence propagation success [84]. Repeated subculturing in in vitro, mound layering, severe pruning and etiolation can rejuvenate stock plants and improve rooting. Results showed that withholding irrigation three to four weeks before taking cuttings increased rooting (56%) relative to the standard irrigation treatment (39%) [83]. These results are confirmed by Niu et al. [85] because in cucumber cuttings under osmotic stress, superoxide has been shown to increase root number per explant when coupled with nitric oxide.

Source of semi-hard wood cuttings affects rooting of walnut. Rooting rate achieved on ‘Paradox’ clone ‘Vlach’ was 42% when stock plants came from tissue culture vs. 78% when stock plants were generated from hardwood cuttings [83]. Rooting percent of semi-hardwood cuttings from field-grown ‘Paradox’ (*J. hindsii* × *J. regia*) increased two times when cuttings were collected from dry stock plants (midday Yw = −1.3 MPa) compared to the same trees six days later upon full hydration (midday Yw = −0.6 MPa) [86].

#### 3.2.5. Rooting Substrate

The substrate used can influence rooting. In rooting hardwood cuttings of Paradox clone ‘Vlach’, the best rooting percentage was obtained using Oasis wedges (81%) compared to rockwool (80%) or sponge plugs (54%) [83]. Among other substrates used for rooting of cuttings and layers are a 2:1 (*v*/*v*) mixture of sawdust (spruce fir) and sand [78] and a 1:1 (*v*/*v*) peat:perlite mix [5], but no detailed comparison has been published.

## 4. In Vitro Propagation

### 4.1. An Overview

The first successful reports of walnut micropropagation date to the 1980s [9,10,87,88,89,90,91]. Genetics, physiological and biochemical condition of explants, environmental conditions and chemical and nutritional composition of the culture medium are among the most important factors involved. Phytohormones and plant growth regulators have significant effects on the ARF of plant species, according to this, in many studies, finding the answer to the use of these substances has been the main goal. In many reports, a wide range of different concentrations of PGRs have been used for root induction, which are summarized in Table 1.

### 4.2. Factors Affecting in Vitro Establishment and Multiplication

In vitro establishment is likely the most unpredictable stage of walnut micropropagation. However, to the best of our knowledge, few studies have been published on this regard. Nonetheless, as with the previous propagation methods, it is determined by various factors. The main drawbacks are the losses caused by microbial contaminations, phenolic releasing and the incapacity of explants to respond to tissue culture conditions. While some genotypes are easy –to culture, others are highly recalcitrant. Differences between clones for Persian walnut [14,111,115,116] and hybrids [111,117] have been observed. Since genotype is a fixed factor that cannot be changed, its effect can be reduced by managing the juvenility degree and culture conditions. Thus, Licea-Moreno et al. [111] (for walnut hybrid) and Yegizbayeva et al. [115] (for *J. regia*) increased the success of in vitro initiation forcing buds to sprout under ex vitro-controlled conditions regarding the use of explants from field-growing trees. Nutritive formulation also has influence on results of in vitro introduction; however, contradictory effects have been obtained. The DKW formulation [9] was created to improve the results of in vitro culture. This has been confirmed by Heile-Sudholt et al. [118], Revilla et al. [89], and Yegizbayeva et al. [115], among others. On the other hand, other authors [11,119] have observed better results using the MS (1962) formulation. This suggests that under some conditions, the selection of culture medium is a key factor to increase the success of in vitro establishment.

The multiplication rate (MR) highly determines the viability of commercial micropropagation. The higher the MRs, the lower the potential production costs. Although general considerations have been made on the improvement of micropropagation, few references exist, in particular, on this stage regarding the increasing of MRs. Nevertheless, some investigations have demonstrated the introduction of some modifications in DKW-C formulation [10], as supplementing it with Phloroglucinol and replacing FeEDTA by FeEDDHA promoted the growth, and the MRs of in vitro materials for walnut hybrids [111] and Persian walnut [115]. Significant differences in MRs of Paradox walnuts [9] American black walnut genotypes [118] and some Persian walnut clones [115] were also registered using DKW medium instead of B5, Cheng, MS and WPM.

Undoubtedly, the introduction of liquid medium would represent a step forward in commercial micropropagation; however, few approaches have been made to date in this regard. Although Heile-Sudholt et al. [118] cultured shoots from American black walnuts in stationary liquid media in their experiments, this did not offer extra information regarding the quality of in vitro materials, nor for MRs. Stevens and Pijut [120] improved the multiplication of two clones of American black walnut using agitated liquid media, but since gelled media were not used as control it is not possible to determine if the complete mortality of rooted microshoots during acclimations was caused by its use or not. Licea-Moreno et al. [110] reported the increase of MRs of various clones of walnut hybrid in temporary immersion bioreactors (TIBs); however, some abnormalities were observed. Later, in 2020, Licea-Moreno et al. [121] solved these problems, producing high-quality microshoots similar to those cultured in gelled media. Nonetheless, more investigations are still needed for the use of temporary immersion systems (TIS) in the commercial micropropagation of walnuts.

### 4.3. Shoot Elongation

Shoot elongation is fundamental to produce adequate explants for rooting. In some species, like olive [122] and walnut [123], a strong dominance of the apical meristem provides the elongation of dominant shoots which naturally occur during the multiplication phase. In such situations, no special elongation media is needed to promote shoot growth, but the composition of the multiplication media, its inorganic salts formulation, the growth regulators used (formulation and concentration), the gelling agents, as well as the origin of the explant used to start the multiplication phase, are factors that may affect the elongation process.

Several auxins and cytokinins have been used for in vitro propagation of walnut, but BAP and IBA have provided the best results, as was suggested by Driver and Kuniyuki [9]. The work of Scaltsoyiannes et al. [14] was fundamental to understanding the role of BAP and IBA in walnut multiplication and shoot elongation. In the absence of IBA, multiplication rate increased with increasing BAP concentration, but shoot elongation was reduced. Adding 0.005 µM IBA to medium containing 2.22 µM BAP achieved an equilibrium between multiplication and elongation. The importance of a low auxin concentration (0.005–0.05 µM) to promote elongation is now generally accepted, but the published results present some contradictions. The most commonly used ratio of cytokinin/auxin is 4.44/0.05 µM.

Other PGRs have been used to stimulate the in vitro growth. Thus, Leslie et al. [106] found 1 mM phloroglucinol had little or no effect on shoot elongation, but enhanced subsequent rooting by an average of 34%. In contrast, Licea-Moreno et al. [110,111] found phloroglucinol was critical for growth of microshoots, but reduced rooting drastically with increasing concentration. 

Saadat and Hennerty [124] reported that shoot elongation is also affected by the type of gelling agent. Phytagel promoted elongation while Difco Bacto agar inhibited it. Some users of DKW medium have avoided Gelrite because of its reputation for promoting hyperhydricity, but Leslie and McGranahan [125] found no problem as long as the culture vessels were not tightly sealed.

### 4.4. Factors Affecting in Vitro Rooting

Unlike the in vitro establishment and multiplication, more investigations have been performed on factors influencing the in vitro rooting. It is recognized as a critical stage of walnut micropropagation because walnuts are usually hard –to root, and the unrooted microshoots have a low probability to survive during acclimatization. Once again, it is highly determined by genotype; however, its influence can be reduced by managing both physical and chemical conditions, as discussed below.

#### 4.4.1. Light

Generally, walnuts are induced to root in the dark for a few days [9,17,83,87,117]. Gruselle et al. [87] did not observe any rooting after 0, 1, 2 and 4 days of darkness, but following 8 and 12 days in the dark, rooting reached 11.1% and 33.3%, respectively. Saadat and Hennerty [99] by culturing ‘Serr’ microshoots in in vitro conditions, also obtained the best rooting percentage (83.35%) with 9 days in the dark (in the root induction stage). Pei et al. [104] reported better rooting (75.4%) in 0 h light than in 16 h (0%), and they also showed that those shoots induced in the dark had more IAA than those in the light. These results are in agreement with those obtained in *Arabidopsis thaliana*, for which the inability to root was correlated with the presence of light and the deregulation of auxin homeostasis, especially in the apices [126].

#### 4.4.2. Temperature

Temperature can affect AR by influencing water uptake and nutrient metabolism and promoting or inhibiting enzymatic action [127]. There is little research on the effect of temperature on in vitro walnut rooting [128]; however, some coincidences have been reported. Rooting of *J. nigra* × *J. regia* ‘A35’ was greater when induced at 22 °C (60%) than at 27 °C (45%) [102]. Similarly, Vahdati et al. [17] found that induction at 22 °C resulted in greater rooting than at 30 °C in ‘Sunland’ (94% vs. 50%), ‘Chandler’ (42 vs. 13%) and ‘Vina’ (25 vs. 3%). In general, temperatures in the range of 20–28 °C have been useful for walnut root induction [107].

#### 4.4.3. Genotype

In vitro response is highly dependent on walnut genotype [10,11,91]. Since the protocols described in the literature are mostly general, great variation in rhizogenesis between genotypes is common (Table 1). However, contradictory results have been obtained regarding the exact relationship between genotypes and root formation [129,130]. Chenevard et al. [94] reported variations in rhizogenesis between two clones of hybrid *J. nigra* No.23 × *J. regia*. Similar variation was found among genotypes of *J. regia* and *J. regia* × *J. nigra* [102]. *J. regia* selections for wood production showed great differences in rooting percent and root length [14]. Although some treatments (such as disbudding and addition of flavanols to the culture medium) improved rooting, results were still largely dependent on genotype [131]. Navatel and Bourrain [100] tried to reduce genotypic effect among Persian walnut varieties by manipulating the physical conditions of root expression medium, but found it caused even greater variations. Recent research did not find statistic interactions between genotype, the culture media used and the site of execution [115], highlighting the importance of genetic factors on rooting ability. 

For ‘Chandler’, some authors have reported similar rooting percentages (ranging from 24.0–33.33%), suggesting a high genetic determinism [20,108,109,112,132]. However, for this variety, some others have reported higher rooting percentages ranging from 55% [17] to 60.5% [104], pointing to the influence of non-genetic factors, such as chemical, physical and even human factors. Yegizbayeva et al. [115] found differences both among Persian walnut genotypes and laboratories for rooting ability, but interactions between both factors were not observed. Other components of rhizogenesis beyond rooting success are also determined by genotype. Licea-Moreno et al. [111] rooted nine clones of hybrid progeny of Mj209 × Ra in vitro and registered variations in rooting rates (63.3–95.0%), root length (17.8–33.3 mm) and mean number of roots per microshoot (3.0–6.1). Zarghami and Salari [112] evaluated rooting of Persian walnut cultivars and found that ‘Chandler’ rooted more frequently (33.33%) and with greater root number (3.16) than ‘Hartley’ (19.44% and 2.1) or ‘Z60′ (19.44% and 1.91). 

There are discrepancies regarding which species and genotypes are the most difficult to root. Hence, Gruselle and Boxus [11] using the same culture conditions, obtained better rooting success (64.8%) and more roots per microshoot (4.22) for *J. regia* than for clones of hybrid ‘Paradox’ (46.4% and 2.9, respectively). In contrast, Dolcet-Sanjuan et al. [102] observed that clones of walnut hybrids (*J. nigra* × *J. regia*) rooted more easily than those of *J. regia*. However, Jay-Allemand et al. [15] reported that clones of *J. major* and hybrid of *J. sieboldiana* × *J. regia* had higher rooting percentages than those of *J. nigra*, although there were also striking differences between the *J. nigra* clones. The low reproducibility of in vitro protocols makes it highly complicated to compare results from different laboratories, even in cases where the same genotypes and variables have been assessed and the same protocols have been followed [115]. 

#### 4.4.4. Culture Medium and Its Components 

The most important media for walnut have been the Driver and Kuniyuki medium (DKW) [9] as modified by McGranahan et al. [10] and the Murashige and Skoog medium (MS) [133] (Table 1). Although DKW is the most widely used for walnut rooting [10,11,17,88,117,134], the MS medium has also provided good results for some genotypes [11,89,100,135,136]. However; Yegizbayeva et al. [115] showed, for four Persian walnut varieties, that DKWC was superior to MS with regard to rooting percentage and the number of roots formed per microshoot. Some researchers have used alternative formulations for Persian walnut. Ashrafi et al. [137] developed a medium that promoted the in vitro growth of ‘Sunland’ and ‘Chandler’, but root formation of both varieties was reduced relative to DKW. Kepenek and Kolağasi [113] used NGE medium, developed by Sánchez-Zamora et al. [114] for walnut embryo germination, and obtained greater than 60% rooting of all genotypes assessed, but did not use any of the more frequently used media as a comparison.

Concerning the concentration of the components added to the culture medium for rooting of walnuts, the use of more dilute formulations has offered better results [103]. Whereas macronutrients are usually reduced to 50% or 25% of full strength for both sub-stages of rooting, some researchers do not alter the micronutrients and vitamins concentrations (see Table 1).

**Figure 1:**
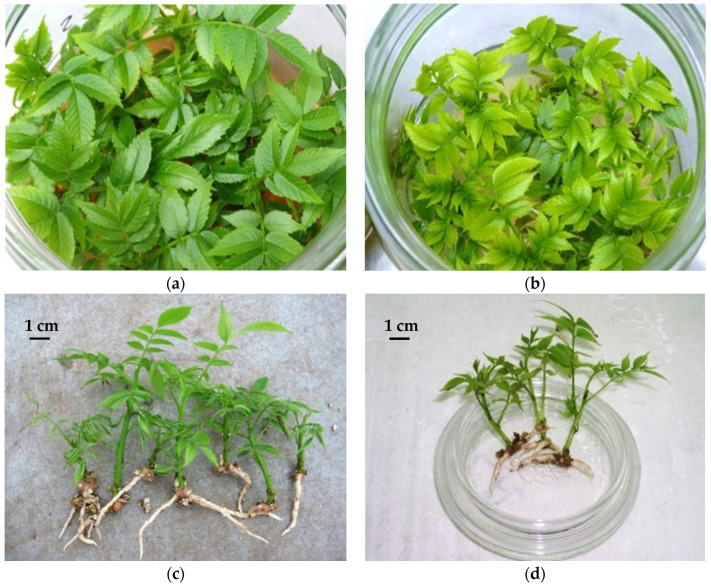
Effect of substituting FeEDDHA (**a**,**c**) for FeEDTA (**b**,**d**) in DKW–C medium, on chlorosis, growth and rooting of clones from the *Juglans* hybrid progeny Mj209 × Ra (photos provided by R.J. Licea-Moreno).

Although major modifications of overall mineral composition (such as those proposed by Ashrafi et al. [137] might impact on rooting, modifications of single nutrients have also had positive effects on ARF. For example, the simple replacement of FeEDTA by FeEDDHA improved rhizogenesis of 8 walnut hybrid genotypes, increasing rooting from 26% to 62%, the number of roots per microshoot from 1.7 to 2.4, and the length of the largest roots from 14.5 mm to 21.9 mm [111]. Microshoots of these clones grown in an excess of FeEDTA completely failed to form roots [111]. However, shoots cultured on lower amounts of iron in the form of FeEDDHA (6.81 mg/L of Fe^3+^ content) exhibited better growth than those on the control using FeEDTA (10.21 mg/L of Fe^2+^ content), and rooting increased from 8.2% up to 77.0% for some clones. Changes in morphology of root systems were also associated with the use of alternate iron sources (Figure 1) [111].

The carbon source may act as either a modulator [138,139,140] or a nutritional factor [139]. Thus, for walnuts it has been demonstrated that either the carbon source or concentrations play key roles in rhizogenesis [94,141,142,143,144]. The concentration of sucrose during proliferation on subsequent walnut rooting has been investigated. Driver [145] found that the concentration of sucrose was among the most important factors assessed for rooting of ‘Paradox’ hybrid walnuts, with a relationship between it and the length of dark treatment and the levels of IBA used. Most protocols include a root pre-induction medium (RPM) containing 87.6 mM sucrose; however, some authors used different doses. Thus, whereas Driver [145] and McGranahan et al. [10] recommended 153.8 mM, Scaltsoyiannes et al. [14] used 117 mM of sucrose. On the contrary, Pijut [97] reduced the sucrose content at this stage to 43.8 mM, but did not offer any details regarding the advantages of this change. Vahdati et al. [17] found that microshoots of Persian walnut ‘Chandler’ did not root at all when only 29.2 mM of sucrose was included in the root pre-induction medium, but rooting percentages increased significantly when concentrations above 87.6 mM were used. Like Scaltsoyiannes et al. [14], Licea-Moreno et al. [110] obtained the greatest root formation for clones of hybrid progeny Mj209 × Ra using 117 mM of sucrose in RPM. The number and length of roots had a positive relationship with increasing concentration. Hence, in general, relatively high sucrose concentration in the RPM medium (between 117 mM and 153 mM) gives the best rooting success.

More attention has been paid to the carbon source used in the root expression subphase. Sucrose is by far the most used carbon source (Table 1), maintaining the same concentration as in proliferation stage. As during induction, the presence of a carbon source is critical for in vitro development of roots. For two *J. nigra* No.23 × *J. regia* hybrid clones, sucrose had a greater effect on rooting than on shoot growth [94]. Rooting percentage, root number and total root length increased dramatically as sucrose concentration increased from 0 to 29.2 mM, with an optimum reached at 117 mM. Microshoots of *J. regia* ‘Serr’ developed on 43.8 mM of sucrose were more prompted to root than on sucrose-free medium, but when sucrose concentration was increased to 87.6, mM rooting percentage was reduced to levels similar to those obtained on no sucrose [102].

**Figure 2:**
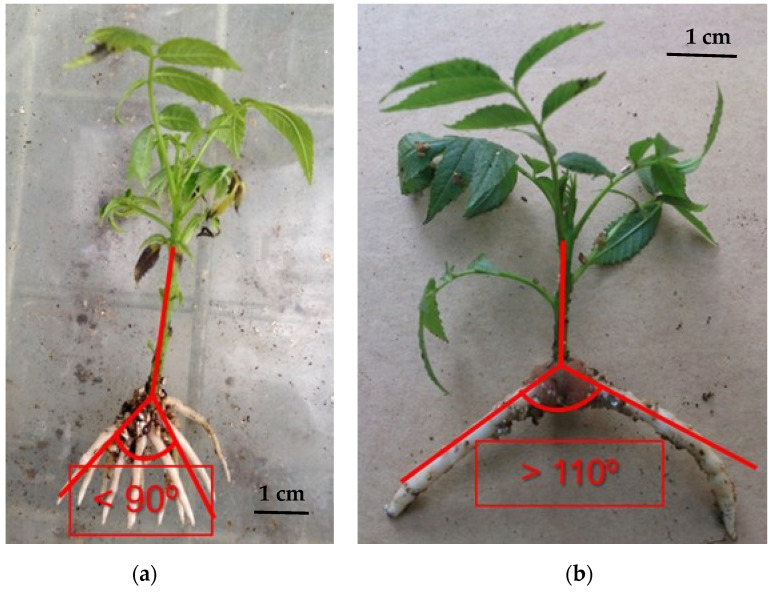

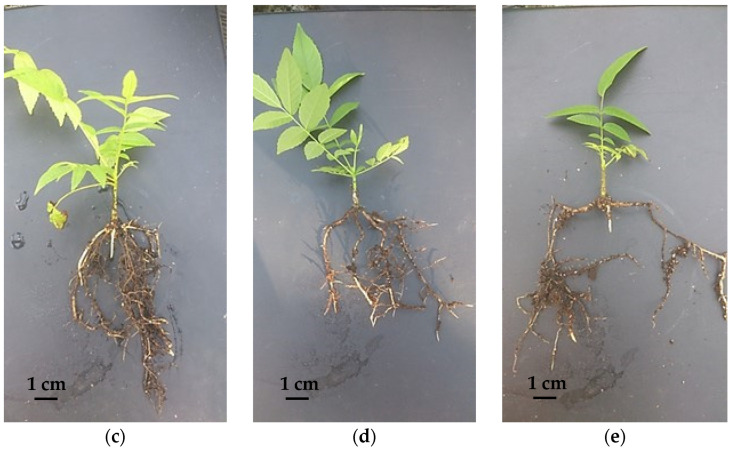
Morphological differences in root systems of *Juglans* hybrid progeny clones of Mj209 × Ra at 3 weeks (**a**,**b**) and 7 weeks (**c**–**e**) after root initiation of microshoots cultured in root expression media supplemented with fructose (**a**,**d**), glucose (**b**,**e**), and sucrose (**c**) (The concentrations used are summarized in Table 1). Photos provided by R.J. Licea-Moreno.

Different carbon sources have shown their ability to influence rhizogenesis of different woody species. Although sucrose has been mostly used during the root expression stage of walnut, other carbon sources have also proved suitable. For rooting hybrid progeny of Mj209 × Ra, Licea-Moreno et al. [110] added fructose into the root-expression culture medium, obtaining 57.4% to 90.9% rooting for the best treatment, depending on genotype. In general, fructose was a better promoter for root formation than glucose or sucrose [111] (Figure 2, Table 1). However, the ex vitro survival was lower for microshoots cultured with fructose than for those on sucrose and/or glucose.

As with iron chelates, carbon source also had a great influence on the morphology of root systems of clones of progeny of Mj209 × Ra (Figure 2, unpublished data). This may have repercussions during transplant and on acclimatization and the success of plant production. Certainly, transplanting microshoots with roots orientated mostly downward could facilitate their manipulation, reducing damage to in vitro-formed roots during transplant and facilitating the use of smaller containers.

Physical support of the culture medium also influences rooting success. Leslie et al. [106] found that gelled culture media are better than liquid. Differences have also been observed between Gelrite and Kobe Agar. Jay-Allemand et al. [15] proposed using vermiculite in the in vitro expression medium to promote root elongation and development of secondary roots. From then on, many authors have used vermiculite, either gelled or not, for species of *Juglans* genre, since it favors root formation (see Table 1).

#### 4.4.5. Age and Quality of Microshoots

Rooting success and survival during acclimatization are highly dependent on the quality of microshoots that are brought to the root initiation stage. For ‘Paradox’, McGranahan et al. [10] recommended using vigorous 3 to 10 cm tall microshoots from multiplication. McGranahan et al. [10] broadened and refined this definition for Persian walnut acclimatization, stating that only those microshoots without necrosis of apical tips and/or defoliation are likely to survive during the acclimatization stage. For clones of hybrid Mj209 × Ra seedlings, Licea-Moreno [146] also stated that healthy and vigorous microshoots with green and abundant leaves were more prepared to root and to overcome the ex vitro conditions during hardening.

To optimize the production of rootable microshoots, Licea-Moreno et al. [110] modified the composition of DKW–C medium and balanced the length of subculture time, the number of explants per container and the volume of culture medium per vessel. In all cases, fewer shoots per container produced longer shoots. When subculture intervals exceeded 6 weeks, microshoots grown at higher densities began to show signs of degradation, e. g., wilting and defoliation, more quickly than those grown at lower density, and rapidly growing microshoots were less able to root, perhaps due to lower accumulation of lignin, as suggested by Bisbis et al. [16]. Thus, it seems that for Mj209 × Ra clones, some degree of lignification is necessary for root initiation, as has been demonstrated for locusts [147]. Driver [145] also hypothesized that some degree of lignification is necessary prior to root induction of hybrid ‘Paradox’. Pei et al. [104] also found that shoot quality strongly affected rooting *J. regia* ‘Xinzaofeng’. Rooting percent of four shoot types varied as follows: (1) vigorous tender shoots with small leaves and long internodes >0.5 cm (83.3%); (2) vigorous with tender leaves and short internodes ≤0.5 cm (71.9%); (3) semi-lignified with broad dark green leaves and long internodes >0.5 cm (7.4%); (4) semi-lignified, often with broad etiolated leaves at the base of the shoot (0%). The quality of the shoot type may reflect their biochemical activities, juvenile status, sensitivity to PGRs or flexibility for cell differentiation.

#### 4.4.6. Shoot Size

Smaller shoots could be produced more rapidly and in greater numbers but larger shoots acclimated and survived more successfully in the greenhouse. However, clones of Mj209 × Ra progeny did not exhibit a clear relationship between microshoot length and rooting [111]. However, Pei et al. [104] found that tender shoots with short internodes (>0.5 cm) and small leaves had the best rooting (83.3%), while semi-lignified shoots with etiolated leaves at the shoot bottoms failed to root at all.

#### 4.4.7. Physical State of Root Expression Media: The Use of Liquid Cultures

Gelling agents differ in their properties and consequently differ in their effects on in vitro cultures. Leslie et al. [106] found that gelled medium mixed with vermiculite produced far better results than stationary liquid medium for rooting several walnut genotypes. However, liquid cultures, either stationary, agitated or in temporary immersion, could improve efficiency of commercial production. Liquid systems are less labor-intensive than production on gelled media and could possibly produce in vitro plants of higher quality, potentially reducing production costs for in vitro plants [148,149,150]. However, both biological and technological problems must first be addressed.

**Figure 3:**
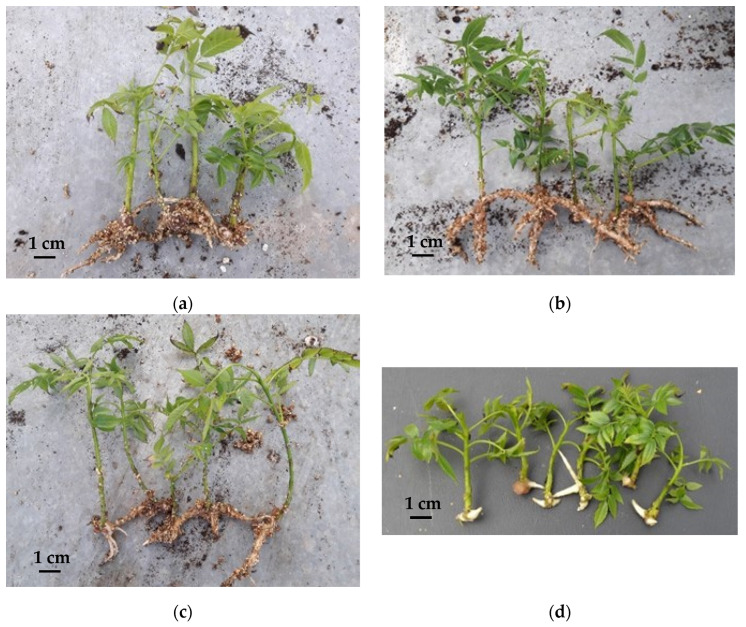
Root expression of a Mj209 × Ra clone in glass containers using: (**a**–**c**) stationary liquid media with vermiculite and (**d**) a temporary immersion system (TIS) using SETIS bioreactors. Microshoots obtained from cultures proliferated in: (**a**,**d**) gelled media, (**b**) TIS, using handmade temporary immersion bioreactors (TIB) and (**c**) Plantform bioreactors (Photos provided by R.J. Licea-Moreno).

Alternative applications of liquid systems for rooting could include: (1) using it only during the expression phase; (2) production of rootable shoots in liquid media, followed by rooting in semi-solid media; (3) both proliferation and rooting performed in liquid systems (stationary, agitated or temporary). The first alternative was used to root clones of Mj209 × Ra hybrids in liquid medium (Figure 3a–d), with either vermiculite (Figure 3a) or a temporary immersion system (TIS) (Figure 3d) as support [110,111]. Liquid medium plus vermiculite resulted in high rooting percentages and the production of high-quality plants that acclimated well [110]. However, the proper ratio of culture medium to vermiculite was necessary to avoid inhibition of rooting, asphyxia of formed roots and hyperhydricity, which increased mortality during the ex vitro acclimation. Mj209 × Ra progeny clones could also be rooted using TIS (unpublished results) but this resulted in low rooting percentages and fewer roots per microshoot. Additionally, morphological changes of formed roots were observed. The most striking was modification of tropism (Figure 3d), making manipulation of the vitroplants difficult during transplanting.

The second alternative was employed by Stevens and Pijut [120]. The authors multiplied two *J. nigra* clones in an agitated liquid system and then obtained 40% rooting in gelled media with vermiculite. However, this was not compared with a control or a reference treatment, and plants did not survive when transferred to a greenhouse. Using the third alternative, clones of Mj209 × Ra progeny were proliferated in several TISs, and roots were developed in liquid media with vermiculite [121]. Type of bioreactor, volume of culture medium and explant type determined rooting success. Root form and quality were improved (Figure 3b,c). Resulting plants were successfully acclimated and grown in the field.

### 4.5. Somatic Embryogenesis

Somatic embryos are not only important for vegetative propagation of walnut, but also for using as a target tissue for gene transfer [10]. Thus, the maintaining of genetic identity of genotypes regenerated from in vitro embryos are of great concern. As previous methods and techniques described, walnut is also recalcitrant for somatic embryogenesis.

Although the first attempts to obtain in vitro embryos were made as early as the 1980s, there are no reports of successful somatic embryogenesis from vegetative tissues in *Juglans* genre, and all of the reports are only from sexual tissues. Hence, most studies have obtained embryos from cotyledons [97,151,152,153,154]. Other explant sources from sexual origins have also been used with different degrees of success, such as endosperm [152], immature zygotic embryos [155] and anthers [97,156].

Regardless of the explant used, it has been reported that similar factors affect the formation and germination of embryos. Like micropropagation from axillary buds, genotype plays an important role. Thus, while Cornu [157] reported embryogenesis of trees from various species in *Juglans* genre and hybrids, other authors have reported differences during the embryogenesis process for some early maturing trees, such as ‘Lara’ variety [155]. The age of explant source has been recognized as a critical factor for callus and embryo formation of *J. nigra* [154] and *J. regia* [97,152,157]. The selection of suitable nutritive formulations and hormones, as well as the use of different light conditions and low temperature treatments, among other factors, have had a great influence on the obtained results. Genetic stability among plantlets obtained by somatic embryogenesis can be evaluated by ISSR and flow cytometry [158].

As with other plant species, in vitro-formed walnut embryos have common bottlenecks, such as the formation of abnormal embryos, lack of synchronicity of maturation and failures of germination. Hence, finding factors involved in embryo formation leads to advances on somatic embryogenesis in the Juglandaceae family.

### 4.6. Acclimatization

In vitro establishment and acclimatization of in vitro produced plants are the greatest challenges of walnut’s micropropagation. Although few studies on walnut acclimatization have been performed, it has been proven that the quality of microshoots, stomatal characteristics and the water conservation capacity of in vitro walnut plants play key roles during walnut hardening [17,159,160]. Nevertheless, because of the reduced capacity of walnut for rooting, only rooted microshoots have the possibility to survive [10,146], although this condition is not always sufficient to ensure survival. In addition to the rooting of the microshoots, the presence of healthy and abundant leaves is necessary to overcome this stage [10,115,146].

It is frequently assumed that stomata produced in vitro are not functional, being the main cause of mortality during acclimatization. However, with the micropropagation protocol performed for reproduction of clones from hybrid progeny Mj209 × Ra, it was possible to obtain non-abnormal stoma, similar to those of adult trees [146], although the formation of stomates is needed to reach their complete functionality. Thus, in general, high relative humidity (>80%) during the first two weeks is necessary. From then on, the reduction of relative humidity contributes to the strengthening of the surviving microshoots. It is also advisable to avoid the direct incidence of sunlight and the occurrence of low temperatures (<16 °C) during the initiation of acclimatization.

### 4.7. Application of Machine Learning

Machine learning (ML) tools allow researchers to perceive the studied process and make proper decisions to develop optimal culture media. Recently, predictive modeling of Persian walnut (*Juglans regia* L.) in in vitro proliferation media using machine learning approaches was performed. In this study, a comparative study was conducted among ANN, KNN and GEP models. To our knowledge, this study is the first application of neural networks and machine learning for optimizing walnut tissue culture media. This technique needs to be used more in future [161].

## 5. Conclusions

Plant breeding methods are successful and efficient if the modified plants have the ability to be propagated vegetatively. Hence, the knowledge of vegetative propagation is as important as the breeding operation itself; therefore, considering the difficulty of vegetative propagation of walnut, it is very important to collect different data from different methods of vegetative propagation of walnut. At present, traditional walnut grafting is a reliable method for walnut propagation and is influenced by environmental and internal factors, while hypocotyl, epicotyl and micro/mini-grafting techniques have been introduced as highly efficient methods, but the optimization of these methods needs more research. The cuttings and stool layering are cheap methods for walnut propagation; however, there is no significant success in these propagation methods, and they are not commercial. Micropropagation is considered the most attractive propagation method in walnut, with the ability to produce countless plants, starting with minimal explant quantities, in a short period of time (around a year). Currently, only a few prominent companies and researchers worldwide have the protocols and the ability to reproduce walnuts by tissue culture. Various factors are involved in the success of tissue culture production, and even after the tissue culture production of walnut culture, the most critical problem is to adapt them to the field conditions. Because micropropagation via axillary buds usually gives low multiplication rates, propagation through somatic embryogenesis is the most suitable alternative for commercial propagation of outstanding genotypes. However, it is constrained to the use of sexual tissues, with further improvements from somatic tissues being necessary.

## Figures and Tables

**Table 1:** In vitro rooting procedures used for walnut species and hybrids, including dark (pre-induction) and light (root expression) phases.

Species	Genotype	WM (w)	Culture Medium	DD (d)	PGR/Dose (µM)	Carbon Source/ Dose (mM)	Culture Medium	Carbon Source/ Dose (mM)	CMS	Root (%)	No. Roots	Reference
*J. regia*		?	MS	0–12	IBA/10	Suc/87.6	MS + IBA	Suc/ 87.6	Gelled	6–100	0–33.3	Gruselle et al. (1987) [87]
*J. major*		?	DKW: ¼Macro x1Micro	5	IBA/24.6	Suc/87.6	DKW: ¼Macro x1Micro	Suc/ 87.6	Gelled vermiculite/perlite	6–100	1.0–8.1	Jay-Allemand et al. (1992) [15]
*J. nigra*												
*J. intermedia*												
*J. sieboldiana × J. regia*												
*J. regia*	RG1, RG2	5	x1MS	7	IBA/15	Suc/87.6	MS: ¼Macro x1Micro	Suc/ 87.6	Gelled vermiculite/Agar	42–96	1.5–4.4	Navatel and Bourrain (1994) [92]
*J. regia*	RG1	?	DKW MS	?	IBA/15	Suc/87.6	DKW: ¼Macro x1Micro	Suc/ 87.6	Gelled vermiculite	0–96	–	Ripetti et al. (1994) [93]
*J. nigra* No.23 *× J. regia*		?	¼DKW	?	IBA/24.6	Suc/87.6	DKW: ¼Macro x1Micro	Suc/ 0–117	Gelled vermiculite/Agar	10–100	1.0–6.0	Chenevard et al. (1995) [94]
*J. regia*	Serr, MB–T–231 **	4	DKW: ¼Macro x1Micro	5	IBA/25	Suc/87.6	DKW: ¼Macro x1Micro	Suc/ 87.6	Gelled vermiculite	5–80	2	Dolcet-Sanjuan et al. (1996) [95]
*J. regia*	RG1	?	MS	7	IBA/15	Suc/43.8	DKW: ¼Macro x1Micro	Suc/ 87.6	Gelled vermiculite	100	–	Heloir et al. (1996) [96]
*J. cinerea*		?	½MS	7; 14; 21	IBA/0–24.6	Suc/43.8	½MS	Suc/ 58.4	Gelled	75	–	Pijut (1997) [97]
*J. regia*	Serr	5–7	MS	8	IBA/15	Suc/87.6	DKW: ¼Macro x1Micro	Suc/ 87.6	Gelled vermiculite	75^1^	1.5	Sanchez-Olate et al. (1997) [98]
*J. regia*	Plemiana1 Plemiana2 clones	4	DKW: ¼Macro x1Micro	3; 6	IBA/24.6	Suc/117	DKW: ¼Macro x1Micro	Suc/ 87.6	Gelled vermiculite	35–90	–	Scaltsoyianns et al. (1998) [14]
*J. regia*	Serr	?	DKW: ¼Macr x1Micro	7	IBA, NAA/10; 15; 20; 25	Suc/87.6	DKW: ¼Macro x1Micro	Suc/ 87.6	Gelled vermiculite	0–37.5	1.0–5.0	Saadat and Hennerty (2001) [99]
*J. regia*	RG2, RG12, RG15, Chandler, Franquette, Lara	5	x1MS	6; 11	IBA/7.5; 25	Suc/87.6	MS: ¼Macro x1Micro	Suc/ 87.6	Gelled vermiculite	45–90	2.0–4.2	Navatel and Bourrain (2001) [100]
*J. regia*	Bansyun, Nan-an, Seiko	5	DKW: ¼Macro x1Micro	5	IBA/25	Suc/87.6	DKW: ¼Macro x1Micro	Suc/ 87.6	Gelled vermiculite	0–47	1.0–2.8	Tetsumura et al. (2002) [101]
*J. regia*		2	¼DKW	5	IAA, IBA, NAA/1; 3.2; 10; 32; 100	?	¼DKW	Suc/ 0; 43.8; 87.6	Gelled vermiculite	5–70	–	Dolcet-Sanjuan et al. (2004) [102]
*J. nigra*												
*J. intermedia*												
*J. regia*	Sunland, Chandler, Vina	4	x1MS	7	IBA/15	Suc/29.2–175	¼DKW	Suc/ 87.6	Gelled vermiculite	0–94	1.4–4.4	Vahdati et al. (2004) [17]
*J. regia*	Sorrento	?	DKW, ½DKW, MS	0; 12; 16; 20	IAA/10; IBA/10; 20 NAA/10	Suc/87.6	DKW	Suc/ 87.6	Gelled	25–58	1.0–3.3	Caboni and Damiano (2005) [103]
*J. regia*	Liaoning, Shangsong, Xinzaofeng, Yuanfeng, Chandler, Vina	3	¼DKW	12	IBA/25	Suc/87.6	¼DKW	Suc/ 87.6	Gelled vermiculite	60.5–87.5	–	Pei et al. (2007) [104]
*J. regia*		?	MS	3	IBA/20	Suc/87.6	¼DKW	Suc/ 87.6	Gelled vermiculite	60.5–87.5	–	Leal et al. (2007) [105]
*J. intermedia*		?	DKW, MS	1–7	IBA/15–50	Suc/87.6	¼DKW	Suc/ 87.6	Gelled vermiculite	0–100	–	Leslie et al. (2005) [106]
*J. regia*		4	¼DKW	4; 7; 14	IBA/50	Suc/87.6	¼DKW	Suc/ 87.6	Gelled vermiculite	24–87	–	Leslie and McGranahan (2009) [107]
*J. intermedia*												
*J. regia*	Sunland, Chandler, Kerman 120	3	½DKW	10	IBA/50	Suc/87.6	¼DKW	Suc/ 87.6	Gelled vermiculite/ Jiffy pot *	0–47	–	Sharifian et al. (2009) [108]
*J. regia*	Sunland, Chandler, Vina	?	1MS	8	IBA/15	Suc/87.6	¼DKW	Suc/ 87.6	Vermiculite	16–501	?	Vahdati et al. (2009) [109]
*J. intermedia*	Mj209 × Ra	4; 5; 6	DKW–C: ½Macro x1Micro FeEDDHA	5	IBA/25; 50	Suc/58; 117; 175	DKW–C: ½Macro, x1Micro, FeEDDHA	Fruc/ 83.2	Liquid + vermiculite	45.3–90.9	1.8–4.7	Licea-Moreno et al. (2012) [110]
*J. intermedia*	Mj209 × Ra	6	DKW–C: ½Macro x1Micro FeEDDHA	5	IBA/50	Suc/117	DKW–C: ½Macro, x1Micro, FeEDDHA	Fruc, Gluc, Suc/ 83.2	Liquid + vermiculite	48.5–95.0	2.1–6.1	Licea-Moreno et al. (2015) [111]
*J. regia*	Chandler, Hartley, Z60	?	DKW	7	IBA/0; 15; 20	Suc/87.6	¼DKW	Suc/ 87.6	Vermiculite	0–41.67	1.49–2.27	Zarghami and Salari (2015) [112]
*J. regia*	Chandler, Kaplan–86, Yalova–1	6	NGE^2^ + PVP^3^	10	IBA/0; 5; 12; 25 + TDZ/0; 4; 11; 23	Glu/222	NGE^1^ + PVP + IBA	Glu/ 222	Phytagel	13.4–75.4	2.2–4.2	Kepenek and Kolağasi (2016) [113]

* WM: Weeks in multiplication before root initiation, DD: Dark period (d), PGR: Plant growth regulator, CMS: Culture medium state (type of gelling agent), * without culture medium. ** also included several clones from zygotic embryo-derived plantlets. ^1^ These data refer to the best media formulation used for proliferation stage. ^2^ Media formulation developed by Sánchez-Zamora et al. (2006) [114]. ^3^ This procedure was developed in a vessel specifically designed for rooting by Kepenek and Kolağasi (2016) [113].
